# Artificial intelligence in breast imaging: Current situation and clinical challenges

**DOI:** 10.1002/EXP.20230007

**Published:** 2023-07-20

**Authors:** Chao You, Yiyuan Shen, Shiyun Sun, Jiayin Zhou, Jiawei Li, Guanhua Su, Eleni Michalopoulou, Weijun Peng, Yajia Gu, Weisheng Guo, Heqi Cao

**Affiliations:** ^1^ Department of Radiology Fudan University Shanghai Cancer Center Shanghai China; ^2^ Department of Oncology Shanghai Medical College Fudan University Shanghai China; ^3^ Department of Breast Surgery Key Laboratory of Breast Cancer in Shanghai Fudan University Shanghai Cancer Center Shanghai China; ^4^ Technical Services Team University of Nottingham Nottingham UK; ^5^ Department of Minimally Invasive Interventional Radiology Key Laboratory of Molecular Target and Clinical Pharmacology School of Pharmaceutical Sciences and The Second Affiliated Hospital Guangzhou Medical University Guangzhou China; ^6^ Department of Health Sciences National Natural Science Foundation of China Beijing China

**Keywords:** artificial intelligence, breast cancer, breast imaging database, deep learning, imaging, national natural science foundation

## Abstract

Breast cancer ranks among the most prevalent malignant tumours and is the primary contributor to cancer‐related deaths in women. Breast imaging is essential for screening, diagnosis, and therapeutic surveillance. With the increasing demand for precision medicine, the heterogeneous nature of breast cancer makes it necessary to deeply mine and rationally utilize the tremendous amount of breast imaging information. With the rapid advancement of computer science, artificial intelligence (AI) has been noted to have great advantages in processing and mining of image information. Therefore, a growing number of scholars have started to focus on and research the utility of AI in breast imaging. Here, an overview of breast imaging databases and recent advances in AI research are provided, the challenges and problems in this field are discussed, and then constructive advice is further provided for ongoing scientific developments from the perspective of the National Natural Science Foundation of China.

## Graphical abstract

This paper provides an overview of the current state of artificial intelligence (AI) in breast imaging, encompassing breast imaging databases, deep learning algorithms, and clinical research. The authors additionally discuss the progress of breast imaging AI through the lens of National Natural Science Foundation of China project proposals. Finally, the authors address the perspectives and challenges in this research domain.

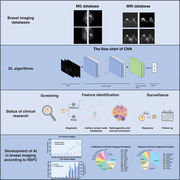

## 1 INTRODUCTION

Breast cancer is a prevalent malignant tumour that endangers the well‐being and survival of women globally, and its incidence is increasing yearly. According to the latest global cancer burden data released by the International Agency for Research on Cancer of the World Health Organization in 2020, breast cancer has the highest morbidity and mortality of all cancers worldwide.^[^ ^1^ ^]^ Breast imaging significantly contributes to breast cancer management to reduce mortality.^[^ ^2^ ^]^

Breast cancer is a heterogeneous disease originating from the mammary epithelium, and subgroups of breast cancer with different molecular characteristics have different prognoses, recurrence and metastasis patterns, and sensitivity to chemotherapy.^[^ ^3^ ^]^ Therefore, it is difficult to conduct a comprehensive tumour assessment based on a physician's personal experience to guide follow‐up treatment. In addition, considerable job burnout among radiologists is associated with the large quantity of imaging data generated daily.^[^ ^4^ ^]^ Therefore, there is an urgent need for a more convenient and efficient workflow.

**FIGURE 1:**
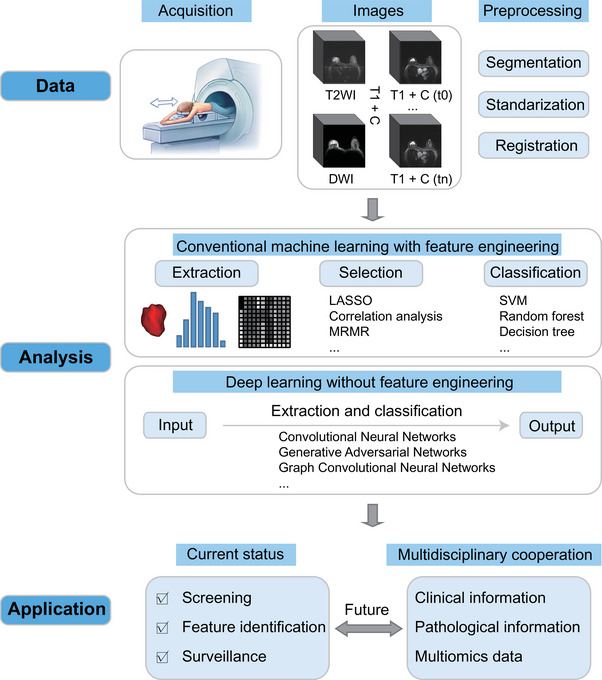
Pipeline of AI‐based breast imaging. LASSO: Least absolute shrinkage and selection operator; MRMR: max‐relevance and min‐redundancy; SVM: support vector machine.

In recent years, artificial intelligence (AI) driven by deep learning (DL) and, in particular, convolutional neural networks (CNNs) has emerged in the field of medical imaging, providing solutions to the challenges in breast imaging described above. AI is suitable for processing repetitive workflows and high‐throughput data and can improve the efficiency of breast imaging and image interpretation.^[^ ^5^ ^]^ In addition, with the development of radiogenomics,^[^ ^6^ ^]^ transcriptomics,^[^ ^7^ ^]^ and metabolomics,^[^ ^8^ ^]^ these highly dimensional complex data can be processed through AI, which has unique advantages in assessing the heterogeneity of breast cancer and providing more possibilities for multidimensional exploration of the pathophysiological mechanism of breast cancer in precision medicine (Figure 1). Here, we review the present status of AI in breast imaging domestically and worldwide in the recent decade, including breast imaging databases, DL algorithms, and clinical research, and further discuss the development of breast imaging AI from the perspective of National Natural Science Foundation of China (NSFC) project proposals. Then, we present the perspectives, problems, and challenges associated with this research field.

## 2 BREAST IMAGING DATABASES

The development and testing of AI‐assisted detection or diagnosis systems for breast cancer require extensive data. By collecting massive images from millions of patients to create a database and integrating multisource data streams such as clinical, pathological, and genetic data to build a complete multidisciplinary data system, AI helps explore the correlation between multisource heterogeneous data and build a multidimensional comprehensive diagnosis and treatment system for breast cancer. The Cancer Imaging Archive (TCIA),^[^ ^9^ ^]^ funded by the National Cancer Institute (NCI) cancer imaging program, is currently the largest open‐access imaging database available. Among the 45 cancer imaging collections in the TCIA, breast cancer accounts for the largest proportion, with 18 public collections. The datasets are mainly based on patients from Europe and the United States and contain only a small number of Asian patients. The TCIA‐Breast dataset contains four modalities: mammography (MG), magnetic resonance imaging (MRI), computed tomography (CT), and positron emission tomography (PET). Among them, MG and MRI are the two major modalities with the largest number of cases and are the most widely used. Below is a concise explanation of these two modalities' datasets.

### 2.1 MG database

Currently, there are six publicly available MG datasets, and each dataset contains normal breasts and pathologically confirmed benign and malignant lesions (Table 1).

**TABLE 1:** MG datasets.

		Imaging modality			Clinical/Pathological information	
Database	Number	FFDM	DM	DBT	Clinical information	Pathological information
MIAS	161	N	Y	N	Y	N
INbreast	115	Y	N	N	Y	N
DDSM	1,566	N	Y	N	Y	Y
CMMD	3,728	N	Y	N	Y	Y
BCS‐DBT	5,060	N	N	Y	Y	Y
VICTRE	2,994	N	N	Y	Y	Y

Y: yes; N: no.

#### 2.1.1 The Mammographic Image Analysis Society (MIAS) database and the INbreast dataset

MIAS is the earliest publicly available mammography dataset. This dataset includes 322 digital medial lateral oblique view (MLO) images of 161 patients, all of which are stored in PNG format.^[^ ^10^ ^]^ INbreast is a publicly available full‐field digital mammography (FFDM) dataset that contains 400 images of 115 participants and provides clinical information and Breast Imaging Reporting and Data System (BI‐RADS) classification information for each patient. The greatest advantage of the INbreast dataset is that it has accurate contour annotations, which is helpful for the development and validation of algorithms for lesion morphology.^[^ ^11^ ^]^

#### 2.1.2 The digital database for screening mammography (DDSM)

DDSM is the most commonly used public MG dataset, consisting of 10,239 images of 1,566 participants from the University of South Florida. The DDSM included the MLO position and the cranio‐caudal view (CC) of each patient, as well as the cropped images with the mass and calcification as the region of interest (ROI). The main purpose of DDSM is to provide a standard MG assessment dataset to develop and test computer‐aided diagnostic (CAD) systems for breast cancer screening and decision support. However, some studies have also noted that the accuracy of DDSM may not be suitable for validating existing segmentation algorithms.^[^ ^12^ ^]^

#### 2.1.3 The Chinese mammography database (CMMD)

CMMD, published by the South China University of Technology, is currently the only MG database composed of Asian populations. This dataset contains 3728 images of 1775 Chinese patients and provides clinical information that matches the images and molecular subtypes of 749 patients (1498 mammograms) to facilitate subgroup analysis. This dataset is mainly used to train a Chinese‐based DL model for breast microcalcification diagnosis.^[^ ^13^ ^]^

#### 2.1.4 The breast cancer screening‐digital breast tomosynthesis database (BCS‐DBT)

BCS‐DBT is currently the largest DBT image database, which consists of 22,032 images of 5060 participants provided by Duke University Hospital. This dataset was initially used in a challenge called DBTex2 sponsored by the American Association of Physicists in Medicine (AAPM). The aim of this competition was to examine the breast lesion detecting capabilities of several AI software applications. The BCS‐DBT dataset provides the location, boundary, and size of each candidate lesion as well as the reliability score.^[^ ^13^, ^14^ ^]^

#### 2.1.5 The in silico trial database

The Virtual Clinical Trial for Regulatory Evaluation (VICTRE) dataset was released by the FDA to evaluate the possibility of using DBT as a substitute for digital mammography (DM). This dataset consists of 217,913 images of 2994 subjects and is mainly used to repeat the previously submitted comparative experiments. In addition, it also provides open‐source and free software tools for systematically exploring experimental parameters, including lesion type and size.^[^ ^15^ ^]^

### 2.2 Breast MRI database

There are eight publicly available breast MRI datasets, which mainly include the breast cancer diagnosis and treatment set and the high‐risk lesion diagnosis set (Table 2).

**TABLE 2:** Breast MRI datasets.

			MRI sequence				Examination time			Clinical/Pathological information		
Database	Number	Therapeutic regimen	T1WI	T2WI	DCE	DWI	Baseline	During NAC	After NAC	Clinical information	Treatment response information	Prognostic information
TCGA	139	AT	Y	Y	Y	N	Y	N	N	Y	–	Y
I‐SPY1	222	NAC	Y	Y	Y	N	Y	Y	Y	Y	Y	Y
I‐SPY2	985	NAC	Y	Y	Y	Y	Y	Y	Y	Y	Y	N
DUKE	922	AT and NAC	Y	N	Y	N	Y	N	N	Y	Y	Y
Pilot	64	NAC	Y	Y	Y	Y	Y	Y	Y	Y	Y	Y
QIN	51	NAC	Y	N	Y	Y	Y	Y	Y	Y	Y	N
ACRIN‐6667	984	–	Y	N	Y	N	Y	N	N	Y	N	N
BREAST‐DIAGNOSIS	88	–	N	Y	Y	N	Y	N	N	Y	N	N

AT: adjuvant therapy; Y: yes; N: no; ‐: not applicable.

#### 2.2.1 The cancer genome atlas breast invasive carcinoma (TCGA‐BRCA) data collection

TCGA‐BRCA is the only breast dataset with matched imaging, clinical, pathological, and genetic information, incorporating preoperative MRI images from 139 breast cancer patients receiving adjuvant therapy. Currently, this dataset provides images, tissue slide images, clinical data, biomedical data, and genomics information, and the follow‐up information is continuously updated. This dataset is based on the Cancer Genome Atlas Program, which aims to explore the correlation between breast cancer genotypes, imaging phenotypes, and patient outcomes. Since the samples of the TCGA‐BRCA dataset are collected from multiple locations worldwide, the scanners, manufacturers, and acquisition protocols are also strongly heterogeneous, which may pose a considerable challenge to image registration and DL model building.^[^ ^9^ ^]^

#### 2.2.2 The I‐SPY 1 and 2 datasets

The I‐SPY 1 dataset derived from the ACRIN‐6657 prospective trial includes dynamic‐contrast enhanced MRI (DCE‐MRI) images of 222 patients receiving neoadjuvant chemotherapy (NAC) from multiple research institutions. It aims to test the performance of MRI in predicting treatment response and recurrence risk of patients with stage 2 or 3 breast cancer who are undergoing NAC.^[^ ^16^ ^]^ The I‐SPY 2 dataset, derived from the multicentre study ACRIN‐6698, is currently the largest breast cancer MRI collection, incorporating images from DCE‐MRI and diffusion‐weighted imaging (DWI) of 385 patients undergoing NAC, with the aim of predicting treatment response in breast cancer patients by imaging and molecular analysis.^[^ ^17^ ^]^ Both the I‐SPY 1 and I‐SPY 2 datasets contain three or four MRI examinations and clinical and pathological information for each patient, but I‐SPY 2 does not provide prognostic information.

#### 2.2.3 The Duke breast cancer MRI dataset

The Duke dataset contains baseline MRI images of 922 patients with biopsy‐confirmed invasive breast cancer from Duke University Hospital, including patients receiving adjuvant and multiple neoadjuvant therapies, together with detailed clinical, pathological and prognostic information. The collection provides image annotation of lesion locations based on DCE‐MRI and annotation of ROIs and extracted image features segmented by autonomous software.^[^ ^18^ ^]^

#### 2.2.4 The Pilot and QIN datasets

Pilot and QIN^[^ ^9^, ^19^ ^]^ include 64 and 51 patients receiving NAC, respectively. The difference is that the former provides three or four MRI examinations during NAC, while the latter provides three PET/CT and quantitative MR images.

#### 2.2.5 Other datasets

In addition to the aforementioned NAC collection, TCIA includes other types of breast datasets. The ACRIN Contralateral Breast MR Database is derived from the clinical trial ACRIN‐6667 and includes contralateral breast MR and MG images of 984 patients with confirmed unilateral breast cancer with corresponding clinical data.^[^ ^20^ ^]^ The BREAST‐DIAGNOSIS dataset contains MRI, MG, CT, and PET/CT images of 88 patients, including high‐risk normal cases, intraductal carcinoma in situ, fibroadenoma, and lobular carcinoma in lesion types.^[^ ^9^ ^]^

## 3 APPLICATION OF DL ALGORITHMS

With the rapid development of AI technology, AI‐assisted medical imaging diagnosis and treatment are considered to have great development prospects.^[^ ^21^ ^]^ DL is an important AI topic and has been widely applied in the processing and analysis of medical images since it was proposed by Geoffrey Hinton in 2006.^[^ ^22^ ^]^ There are many reports on the application of DL algorithms in breast image processing and analysis.

### 3.1 Flow of breast image processing and analysis

**FIGURE 2:**
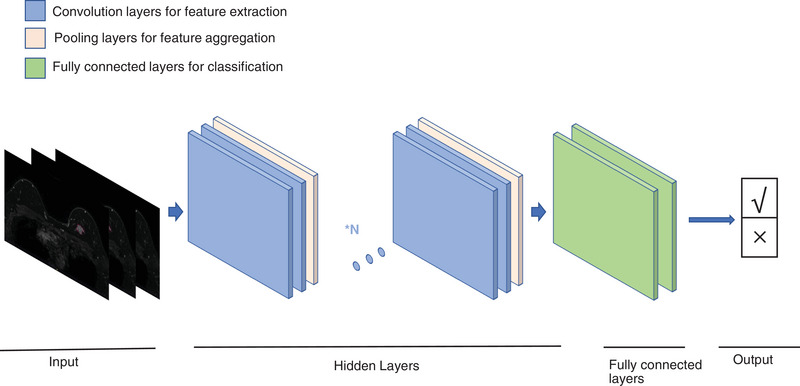
Process of CNN for medical imaging classification.

The flow of breast image processing and analysis includes the following three steps: (1) data acquisition, which mainly includes MG, ultrasound (US), and MRI; (2) image preprocessing, including breast image registration and segmentation; and (3) mining image information of different modalities for analysis and prediction, including image detection and classification. CNNs are one of the most commonly used DL architectures and can be effectively applied to image segmentation, detection and classification.^[^ ^23^ ^]^ A typical CNN includes an input layer, a convolution layer, a pooling layer, a fully connected layer, and an output layer.^[^ ^24^ ^]^ A CNN flow chart with medical image classification as an example is shown in Figure 2. Currently, popular algorithms such as transformer models, generative adversarial networks (GANs), and graph convolutional neural networks are also increasingly used in the analysis of breast images.

### 3.2 Application of DL algorithms in breast image segmentation

**FIGURE 3:**
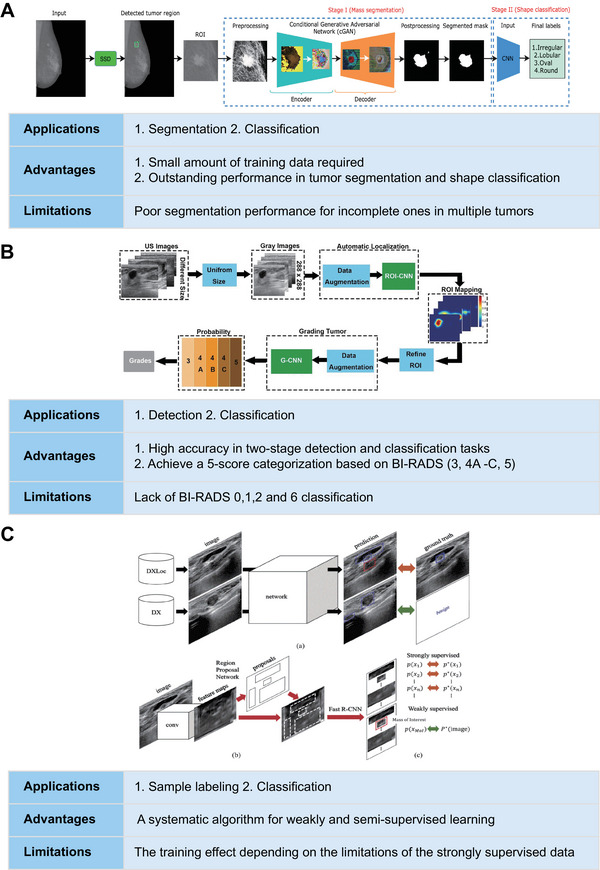
Examples of specific applications, advantages, and limitations of DL algorithms applied in breast imaging. (A) Reproduced with permission.^[^ ^27^ ^]^ Copyright 2020, Elsevier. (B) Reproduced under the terms of the Creative Commons CC BY license.^[^ ^29a^ ^]^ Copyright 2019, Springer Nature. (C) Reproduced with permission.^[^ ^37^ ^]^ Copyright 2019, IEEE.

DL algorithms enable the automatic segmentation of breast tumours in images. Commonly used segmentation models include V‐Net, U‐Net, SegNet, and cGAN. U‐Net effectively blends low‐ and high‐resolution image characteristics by jumping connections, making it the standard approach for numerous medical image segmentation tasks.^[^ ^25^ ^]^ U‐Net integrated with transformer models combines the advantages of convolution and self‐attention strategies to achieve tumour segmentation.^[^ ^26^ ^]^ The convolution layer extracts local intensity features, and the self‐attention mechanism is used to capture global features, thereby improving segmentation accuracy. Vivek et al. proposed a segmentation model based on cGAN, where the generative network was trained to detect tumour regions and generate segmentation outcomes, while the adversarial network learned to distinguish ground‐truth and segmentation outcomes generated by the generative network, thus forcing the generative network to obtain as actual a label as possible (Figure 3A).^[^ ^27^ ^]^

One of the difficulties in segmenting breast tumours is to segment tumour boundaries with blurred borders. Distinguishing the boundaries of nonmass‐enhanced lesions and distinguishing between background parenchymal enhancement and tumours are also key future research directions. Currently, there are many strategies to deal with these problems, such as making full use of the complementary information encoded in CNN's different layers and using the attention mechanism to selectively leverage the multilevel features integrated from different layers to refine the features of each layer, suppressing the noise in the shallow layer and adding more tumour details to the deep layer features.^[^ ^28^ ^]^

### 3.3 Application of DL algorithms in breast image detection and classification

The application of breast imaging clinical analysis mainly focuses on image detection and image classification. The commonly used detection models include YOLO and ROI‐CNN;^[^ ^29^ ^]^ the commonly used classification models include YOLO, GoogLeNet, AlexNet, ResNet, and VGG.^[^ ^29^, ^30^ ^]^ In MG, Aly et al. conducted a study to evaluate the efficacy of the YOLO algorithm for detecting and classifying breast masses.^[^ ^29c^ ^]^ YOLO can detect masses in mammograms and differentiate benign and malignant lesions without human intervention. The team compared the performance of three different YOLO architectures for detection and classification. The highest detection accuracy was achieved when utilizing k‐means clustering to the dataset with the anchor box concept in YOLO‐V3, detecting 89.4% of the masses and distinguishing between benign and malignant masses with accuracies of 94.2% and 84.6%, respectively. In the US, Huang et al. proposed a two‐stage grading system based on CNN. The tumour recognition network ROI‐CNN was constructed to identify the ROI from the original image. The downstream tumour classification network G‐CNN generated valid features to automatically classify the ROI into five categories (3/4A/4B/4C/5) according to BI‐RADS. Its classification accuracy was comparable to that based on the radiologists’ subjective classifications (Figure 3B).^[^ ^29a^ ^]^ Since MRI has a higher spatial and temporal dimension and a higher soft tissue resolution, its research direction is slightly different from that of MG and US. It focuses more on the prediction of outcome and efficacy. Dalmis et al. used the DenseNet algorithm to combine imaging and clinical information to construct a model to identify benign and malignant lesions and achieved an area under the curve (AUC) of 0.852.^[^ ^30b^ ^]^ Braman et al. constructed a multi‐input CNN model for predicting pathological complete response (pCR) of HER2+ breast cancer to NAC. The results were statistically superior to traditional prediction methods, showing the potential of DL for such tasks.^[^ ^31^ ^]^ In addition, GoogLeNet, AlexNet, ResNet, and VGG can be applied to various classification tasks, as shown in Table 3.

**TABLE 3:** Representative DL models for breast image processing and analysis.

Studies	DL model	Imaging modality	Dataset	Image dimensions	Tasks	Model name	Supervision methods
Cai H et al.^[^ ^13a^ ^]^	CNN	MG	Internal dataset	2D	Classification	AlexNet	Supervised learning
Aly GH et al.^[^ ^29c^ ^]^	CNN	MG	INbreast database	2D	Detection	YOLO	Supervised learning
					Classification		
Al‐Antari MA et al.^[^ ^29b^ ^]^	CNN	MG	INbreast database	2D	Detection	YOLO	Supervised learning
					Classification	CNN, ResNet, InceptionResNet	
Kim H‐E et al.^[^ ^30d^ ^]^	CNN	MG	Internal dataset	2D	Classification	ResNet‐34	Supervised learning
Fujioka T et al.^[^ ^30c^ ^]^	CNN	US	Internal dataset	2D	Classification	GoogLeNet	Supervised learning
Huang Y et al.^[^ ^29a^ ^]^	CNN	US	Internal dataset	2D	Detection	ROI‐CNN	Supervised learning
					Classification	G‐CNN	
Kumar V et al.^[^ ^59^ ^]^	CNN	US	Internal dataset	2D	Segmentation	Multi U‐net	Supervised learning
Dalmis MU et al.^[^ ^30b^ ^]^	CNN	MRI (DCE‐MRI, T2WI, DWI, ADC maps)	Internal dataset	3D	Segmentation	DenseNet	Supervised learning
Liu W et al.^[^ ^30a^ ^]^	CNN	MRI (DCE‐MRI, T2WI, DWI)	Internal dataset	3D	Classification	VGG16	Supervised learning
Truhn D et al.^[^ ^60^ ^]^	CNN	DCE‐MRI	Internal dataset	3D	Classification	ResNet18	Supervised learning
Braman N et al.^[^ ^61^ ^]^	CNN	DCE‐MRI	Internal dataset	3D	Classification	Multi‐Input CNN	Supervised learning

Currently, most studies on the classification and detection of breast imaging are based on single modalities and limited indicators. However, since images of different modalities express different information and indicators that need to be comprehensively considered when making clinical decisions, multi‐indicator prediction based on multiparametric and multimodal images is crucial. Fan et al. first proposed a multitask learning framework based on multiparametric MRI fusion to simultaneously predict multiple pathological indicators (histologic grade, Ki‐67) of breast cancer. In the first stage, a multitask feature selection method was employed to simultaneously select feature sets related to all tasks from each image sequence. In the second stage, a multitask classifier was used to construct a model using a convex optimization problem to complete the multi‐indicator prediction. The results demonstrated that the multitask learning method can effectively enhance the prediction performance of a single task, and the prediction effect of the multiparameter image‐integrated prediction model was superior to that of the single‐parameter image model.^[^ ^32^ ^]^ In addition, with the development of new MG‐derived technologies, extending DL from traditional 2D images to tomosynthesis images has become an urgent problem. Zheng et al. replaced the 2D convolutional kernels in the ResNet network model with 3D convolutional kernels and used the 3D‐ResNet network model combined with a migration learning strategy to classify 3D masses, achieving similar classification performance to traditional machine learning and greatly saving operation time.^[^ ^33^ ^]^

Notably, although DL models show considerable promise in two important clinical scenarios, lesion detection and classification, these models still have some disadvantages. For instance, YOLO is not good at detecting small objects close to each other,^[^ ^29c^ ^]^ and AlexNet can only handle relatively small‐sized images.^[^ ^34^ ^]^ In addition, the “black box” problem of DL models is a widespread issue of concern. “Black box” is a situation where, as an outside observer, humans are not informed of how the AI system determines associations as relevancies.^[^ ^35^ ^]^ This is particularly important in clinical scenarios such as lesion detection and classification, which raises ethical and regulatory issues. “Explainable AI” techniques can help understand the predictions of DL models and will help strengthen diagnostic confidence and improve regulatory regimes.^[^ ^36^ ^]^

### 3.4 Application of DL algorithms in sample labelling

Existing medical imaging AI studies suffer from the problem of small samples of high‐quality labelled data, and studies on breast imaging face the problem of high labelling costs and the small size of labelled samples. To solve these problems, many researchers have proposed weakly supervised learning methods, such as combining weakly annotated datasets with small strongly annotated datasets. Shin et al. trained the Faster‐R‐CNN‐based network using weakly supervised learning. Compared to training the same amount of strongly annotated datasets, training both weakly and strongly annotated images can greatly improve performance (Figure 3C).^[^ ^37^ ^]^ Another example is multiple instance learning (MIL), which only needs to annotate the overall medical images. Quellec et al. applied MIL to adaptively segment the breast into multiple regions and then extracted features from each region for classification. The weakly supervised MIL outperformed manual segmentation for classification.^[^ ^38^ ^]^ These methods reduce the training cost and improve the accuracy to some degree, but they still cannot completely extract all the features in the image, which can be further improved through unsupervised learning in the future.

## 4 STATUS OF CLINICAL RESEARCH

### 4.1 Screening

MG is one of the earliest imaging methods combined with AI in breast cancer screening. AI improves screening MG examinations by improving the sensitivity of breast cancer detection, reducing the recall and biopsy, and reducing the interpretation time. The AI models developed in several studies have significantly improved lesion detection and provided additional value for diagnosis. In an AI model developed by large‐scale MG data,^[^ ^30d^ ^]^ AI showed remarkable efficacy in breast cancer detection, with an AUC of 0.940 (0.915–0.965) for automated reading, which was much greater than that of radiologists without the use of AI (0.810, 95% CI 0.770–0.850); with the help of AI, the diagnostic efficacy of radiologists was enhanced to 0.881 (0.850–0.911; *p* < 0.0001). Liu et al. developed a DL model based on MG images of 384 patients combined with clinical factors to predict BI‐RADS 4 microcalcification. The AUC, sensitivity, and specificity were 0.910, 85.3%, and 91.9%, respectively, reaching senior radiologists’ diagnostic performance and surpassing junior radiologists’.^[^ ^39^ ^]^ Yi et al. reviewed BI‐RAIDS 0 MG images of 1,010 recalled patients. They found that with the assistance of the AI system, mid‐level radiologists could effectively reduce the recall rate of BI‐RADS 0 patients and the rate of benign biopsy without missing high‐grade malignant tumours and could reduce the time for junior radiologists to evaluate BI‐RADS 0 lesions.^[^ ^40^ ^]^ Rodriguez‐Ruiz A et al. explored the feasibility of AI to automatically identify normal MG examinations to reduce the workload of breast cancer screening. The results demonstrated that by using a score between 1 and 10 to indicate the likelihood of cancer presence, radiologists could reduce their workload by 47% while excluding 7% true‐positive results when reading only exam images with a score of 5 or more and by 17% when the threshold was 2 while excluding only 1% true‐positive exams.^[^ ^41^ ^]^

In addition, CAD systems based on breast US and MRI techniques show good screening capabilities. Jiang et al. found that when using a DL‐based CAD system, the screening accuracy of automatic breast US for women with dense breast tissue was improved (0.828 vs 0.848), and the scanning time was shortened (3.55 min/case vs 2.4 min/case).^[^ ^42^ ^]^ Since manual analysis is time consuming and subjective, Qi et al. established an automatic diagnosis model based on a deep CNN that can evaluate the existence of malignant tumours in breast US pictures and identify solid nodules, thereby improving the efficiency and reliability of breast cancer screening.^[^ ^43^ ^]^ Illan et al. combined independent component analysis and machine learning to optimize the efficacy of the CAD system in detecting and segmenting nonmass‐enhanced lesions in DCE‐MRI, which effectively reduced the false‐positive rate.^[^ ^44^ ^]^

### 4.2 Feature identification

AI combines radiomics features with clinical features, pathological features, genomics, and other multiomics features, developing from single to multimodality, and explores extensively in differential diagnosis, axillary lymph node prediction, and biological characterization at the microscopic level, such as the molecular or genomic level.

In terms of differential diagnosis, AI focuses mostly on the distinction of benign and malignant lesions and the identification of molecular subtypes of breast cancer. AI models based on DL,^[^ ^45^ ^]^ especially CNNs,^[^ ^30^, ^46^ ^]^ can meet or even surpass radiologists in terms of sensitivity, specificity, and accuracy in identifying benign and malignant lesions. For example, Chougrad et al. used a pretrained CNN model to distinguish benign and malignant lesions on an independent dataset of 113 MG images, with an AUC of up to 0.99.^[^ ^46a^ ^]^ In addition, combining multimodal imaging data to identify the molecular subtypes of breast cancer is also effective. For example, Zhou et al.^[^ ^47^ ^]^ constructed an assembled CNN (ACNN) based on the three modal datasets of grayscale US, colour Doppler flow imaging, and shear wave elastography (SWE) of 818 breast cancer patients. The ACNN preoperative prediction of molecular subtypes (AUC: 0.89–0.96) was better than that of the single‐modality (0.73–0.75) and bimodal (0.81–0.84) models. Ma et al.,^[^ ^48^ ^]^ based on MG and US images of 600 patients with invasive breast cancer combined with clinical features, constructed a machine learning model for molecular subtypes and found that the decision tree model distinguished triple‐negative breast cancer (TNBC) from other breast cancer subtypes most accurately (AUC: 0.971, accuracy: 0.947, sensitivity: 0.905, specificity: 0.941).

Breast US^[^ ^49^ ^]^ and MRI^[^ ^50^ ^]^ techniques are often used to examine axillary lymph node involvement in individuals with breast cancer. Zhou et al.^[^ ^49a^ ^]^ applied CNN models for the first time to predict the likelihood of clinically negative axillary lymph node metastases in primary breast cancer patients based on US images of 756 patients, achieving AUCs of 0.90 and 0.89 in the internal and external test sets, respectively, which performed significantly better than experienced radiologists. Zheng et al.^[^ ^49c^ ^]^ combined conventional US and SWE modalities to develop a DL model for predicting axillary lymph node involvement based on US images and clinical parameters of 584 patients with early‐stage breast cancer. The model performed well in predicting both no axillary metastasis and any axillary lymph node metastasis (AUC: 0.902) and mild and severe metastatic burden (AUC: 0.905), providing a basis for the development of appropriate axillary treatment regimens for early‐stage breast cancer patients. In addition, Yu et al.^[^ ^50b^ ^]^ combined MRI radiomics, genomics, transcriptomics, and clinical pathological information to construct multiomics features in the training set, which can be used to identify patients with axillary lymph node metastasis in early invasive breast cancer before surgery. The AUCs of the training set and the external validation set reached 0.90 and 0.91, respectively.

In addition, radiomics combined with multiomics analysis can also be used to reveal the heterogeneity and microenvironment of breast cancer, which has become a hot topic of research in recent years.^[^ ^51^ ^]^ For example, Jiang et al.^[^ ^51b^ ^]^ quantitatively extracted intratumoural and peritumoral radiomics from the DCE‐MRI images of 202 TNBC patients, combined with transcriptome and metabolomic data, proving that peritumoral heterogeneity is associated with immunosuppression and upregulation of fatty acid synthesis.

### 4.3 Surveillance

Breast cancer surveillance is a longitudinal analysis of tumour changes over time, including response to NAC and prognosis.^[^ ^52^ ^]^ The automated, quantifiable, and repeatable nature of AI technology provides the potential to accurately track lesions.^[^ ^53^ ^]^ In terms of efficacy prediction, AI provides an effective and workable tool for predicting NAC response and determining individualized treatment regimens.^[^ ^54^ ^]^ For example, based on US pictures of 168 breast cancer patients before and after the second and fourth NAC courses, Gu developed DL radiomics (DLR2 and DLR4) models to predict the response after those courses. A new DL radiomics pipeline (DLRP) was proposed by combining DLR2 and DLR4 to gradually predict the response of patients to NAC.^[^ ^54b^ ^]^ In terms of prognosis prediction, several studies have been conducted based on machine learning and radiomics. For instance, Bhattarai S et al. built a machine learning model using two consecutive MG images to predict the in vivo growth rate of tumours.^[^ ^55^ ^]^ Wang HY et al. integrated US‐radiomics features and clinicopathological features to build a machine learning radiomics model to predict disease‐free survival in TNBC with an AUC of 0.90.^[^ ^56^ ^]^ Jiang et al.^[^ ^51b^ ^]^ demonstrated that radiomics characteristics that reflect peritumoral heterogeneity can predict recurrence‐free survival and overall survival in individuals with TNBC and that higher peritumoral heterogeneity indicates poor prognosis and more aggressive tumour characteristics.

**FIGURE 4:**
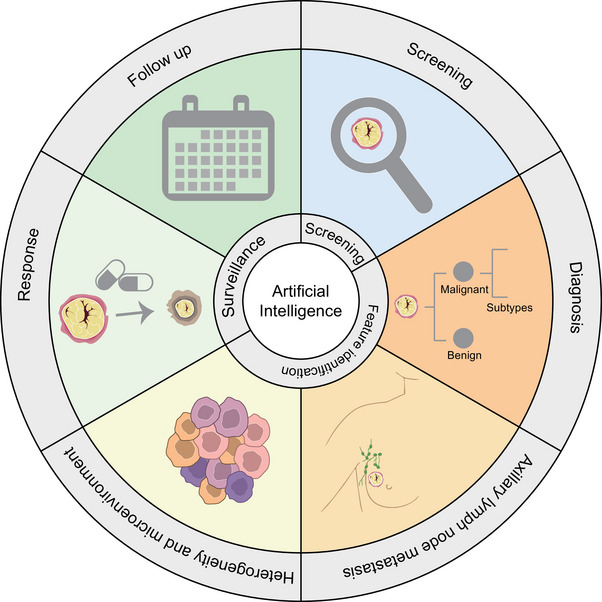
Current status of research on AI imaging in clinical applications of breast cancer.

In summary, AI has been investigated in various modalities of breast imaging (MG, US, and MRI), providing more possibilities for optimizing the early detection of breast cancer, acquisition of clinical features, precision treatment, and efficacy surveillance (Table 4). However, it should be noted that these methods still need to be tested in multicentre and larger‐scale populations; the quality control and risk assessment of AI and the storage and transmission of large amounts of data, as well as the ethical issues that may arise also require further cautious evaluation (Figure 4).

**TABLE 4:** Clinical studies on the application of AI in breast cancer screening, feature characterization, and surveillance.

Study	Imaging modality	Application	Key findings
Kim HE et al.^[^ ^30d^ ^]^	MG	Screening	The AI algorithm showed better diagnostic performance in breast cancer detection compared with radiologists (AUC: 0.940 vs 0.810). Radiologists’ performance improved (0.881) when aided by AI.
Rodriguez‐Ruiz A et al.^[^ ^41^ ^]^	MG	Screening	AI can be used to automatically preselect screening items to reduce the reading workload for breast cancer screening.
Jiang Y et al.^[^ ^42^ ^]^	US	Screening	The deep learning‐based CAD system helped radiologists improve screening accuracy (AUC: 0.848 vs. 0.828).
Qi X et al.^[^ ^43^ ^]^	US	Screening	Mt‐Net and Sn‐Net were proposed to identify malignant tumours and recognize solid nodules in a cascade manner, with an AUC of 0.982 for Mt‐Net and 0.928 for Sn‐Net.
Illan IA et al.^[^ ^44^ ^]^	MRI	Screening	Independent component analysis was used to extract data‐driven dynamic lesion characterizations to address the challenges of nonmass‐enhancing lesion detection and segmentation.
Chougrad H et al.^[^ ^46a^ ^]^	MG	Classification of malignant and benign lesions	A CAD system based on a dCNN model to classify benign and malignant lesions (AUC = 0.99, accuracy: 98.23%).
Akselrod‐Ballin A et al.^[^ ^45a^ ^]^	MG	Classification of malignant and benign lesions	An algorithm combining ML and DL approaches was used to classify benign and malignant masses (AUC = 0.91), with a level comparable to radiologists.
Ciritsis A et al.^[^ ^46b^ ^]^	US	Classification of lesions according to BI‐RADS catalog	dCNNs may be used to mimic human decision‐making in classifying BI‐RADS 2–3 versus 4–5 (accuracy: 93.1% vs 91.6 ± 5.4%).
Han S et al.^[^ ^46c^ ^]^	US	Classification of malignant and benign lesions	The proposed deep learning‐based method had great discriminating performance in a large dataset for classifying benign and malignant lesions (Accuracy: 90%, sensitivity: 0.86, specificity: 0.96).
Fujioka T et al.^[^ ^30c^ ^]^	US	Classification of malignant and benign lesions	Deep learning with CNN showed a high diagnostic performance to discriminate between benign and malignant breast masses on ultrasound (AUC = 0.913 vs 0.728−0.845).
Jiang Y et al.^[^ ^62^ ^]^	MRI	Classification of malignant and benign lesions	The AI system improved radiologists’ performance in differentiating benign and malignant breast lesions on MRI (ΔAUC = +0.05).
Zhou BY et al.^[^ ^47^ ^]^	US	Prediction of molecular subtypes	The multimodal US‐based ACNN model performed better than the monomodal or dual‐modal model in predicting four‐classification, five‐classification molecular subtypes and identifying TNBC from non‐TNBC.
Ma M et al.^[^ ^48^ ^]^	US, MG	Prediction of molecular subtypes	The interpretable machine learning model could help clinicians and radiologists differentiate between breast cancer molecular subtypes, and the decision tree model performed best in distinguishing TNBC from other breast cancer subtypes (AUC = 0.971).
Ha R et al.^[^ ^63^ ^]^	MRI	Prediction of molecular subtypes	A CNN algorithm was developed to predict the molecular subtype of breast cancer based on features on breast MRI images (accuracy: 70%, AUC = 0.853).
Zhou LQ et al.^[^ ^49a^ ^]^	US	Prediction of lymph node metastasis	The best‐performing CNN model, Inception V3, predicted clinically negative axillary lymph node metastasis with higher sensitivity (85% vs 73%) and specificity (73% vs 63%) than radiologists.
Zhang Q et al.^[^ ^49b^ ^]^	US	Prediction of lymph node metastasis	A computer‐assisted method using dual‐modal features extracted from real‐time elastography and B‐mode ultrasound was valuable for discrimination between benign and metastatic lymph nodes (AUC = 0.895).
Zheng X et al.^[^ ^49c^ ^]^	US	Prediction of lymph node metastasis	DL radiomics of conventional ultrasound and shear wave elastography combined with clinical parameters yielded the best diagnostic performance in predicting disease‐free axilla and any axillary metastasis in early‐stage breast cancer (AUC = 0.902).
Ha R et al.^[^ ^50a^ ^]^	MRI	Prediction of lymph node metastasis	The feasibility of using CNN models to predict lymph node metastasis based on MRI images was proved (accuracy: 84.3%).
Yu Y et al.^[^ ^50b^ ^]^	MRI	Prediction of lymph node metastasis	A multiomic signature incorporating key radiomic features extracted by machine learning random forest algorithm, clinical and pathologic characteristics, and molecular subtypes could preoperatively identify patients with axillary lymph node metastasis in early‐stage invasive breast cancer (AUC = 0.90 and 0.91 in the training and external validation sets, respectively).
Jiang L et al.^[^ ^51b^ ^]^	MRI	Tumour microenvironment revelation; prognosis prediction	Peritumoral heterogeneity correlated with metabolic and immune abnormalities in TNBC; radiomic features reflecting peritumoral heterogeneity indicated TNBC prognosis.
Arefan D et al.^[^ ^51a^ ^]^	MRI	Tumour microenvironment revelation	Breast MRI‐derived radiomics extracted by a radio‐genomics approach and machine learning models were associated with the tumour's microenvironment in terms of the abundance of several cell types.
Kavya R et al.^[^ ^54a^ ^]^	MRI	Prediction of NAC response	A CNN model combining pre‐ and postcontrast DCE‐MRI images was proposed to predict which NAC recipients will achieve pathological complete response (AUC = 0.77).
Gu J et al.^[^ ^54b^ ^]^	US	Prediction of NAC response	Deep learning radiomics models were proposed to stepwise predict the response to NAC at different NAC time points.

## 5 DEVELOPMENT OF AI IN BREAST IMAGING IN CHINA ACCORDING TO THE NSFC

The NSFC is a significant directing force in the field of fundamental science and technology innovation in China. In the past decade (2012–2021), the NSFC has closely focused on the “four aspects” of President Jinping Xi's important requirements for China's scientific and technological innovation and provided strong support to the field of medical AI research. In terms of discipline distribution, the Department of Medicine of the NSFC established the discipline of big data and AI in medical imaging (H2709) in 2020 to provide exceptional support to the above fields. In the past decade, the NSFC has approved 295 projects in the field of big data and AI research, of which 29 projects are related to breast imaging, promoting the enthusiasm and extensive attention of many breast imaging researchers in this field.

**FIGURE 5:**
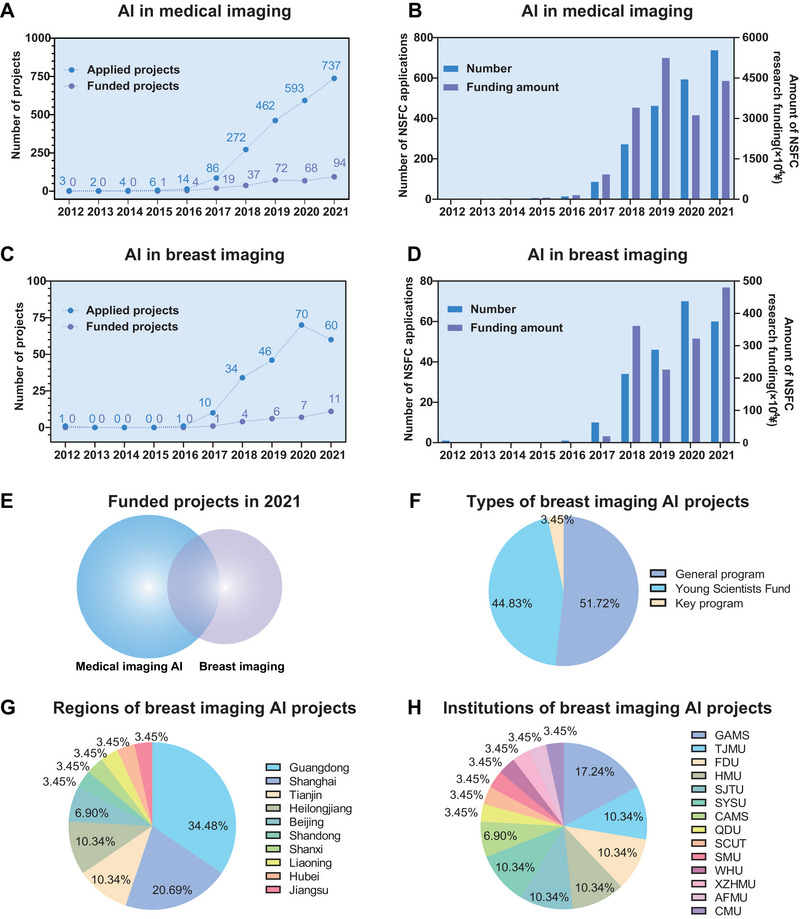
Statistics of the National Nature Science Foundation of China (NSFC) research of AI in medical imaging and breast imaging in the past decade. (A) Annual number of projects in AI in medical imaging applied for and approved by NSFC in 2012–2021. (B) Annual numbers of NSFC applications and amount of research funding for AI in medical imaging from 2012 to 2021. (C) Annual number of projects in AI in breast imaging applied for and approved by NSFC in 2012–2021. (D) Annual numbers of NSFC applications and amount of research funding for AI in breast imaging from 2012 to 2021. (E) Funded project in medical imaging AI and breast imaging in 2021. (F) Types of breast imaging AI projects from 2012 to 2021. (G) Regions of breast imaging AI projects from 2012 to 2021. (H) Institutions of breast imaging AI projects from 2012 to 2021 (GAMS: Guangdong Academy of Medical Sciences; TJMU: Tianjin Medical University; FDU: Fudan University; HMU: Harbin Medical University; SJTU: Shanghai Jiaotong University; SYSU; Sun Yat‐sen University; CAMS: Chinese Academy of Medical Sciences; QDU: Qingdao University; SCUT: South China University of Technology; SMU: Southern Medical University; WHI: Wuhan University; XZHMU: Xuzhou Medical University; AFMU: Air Force Medical University; CMU: China Medical University).

In terms of the number of applications and the amount of funding for medical imaging AI projects, we are embracing the upsurge of medical AI research. Before 2017, the number of applications for AI in medical imaging was negligible. Since 2017, with the gradual maturity of medical AI analysis technology, the number of applications has increased annually (Figure 5A). By 2021, the total number of applications reached 737 cases, which was 8.57 times that in 2017. As the number of applications increased rapidly, the number of funded projects also showed a steady increase. In 2021, the cumulative number of medical imaging AI projects reached 295, of which the number of projects approved in 2021 was the highest, with 94 projects funded. The amount of funding in 2019 was the highest in the last decade, reaching RMB 52.44 million (Figure 5B).

The number of applications and funding for AI in breast imaging also showed an upward trend (Figure 5C). The total number of applications in 2020 was the highest, with 70 applications, which was seven times that in 2017. Although the number of applications in 2021 decreased slightly, the number of funded projects in AI in breast imaging increased year by year. In 2021, there were 11 projects approved in the direction of AI in breast imaging (compared to one in 2017), with the highest funding in the last decade at RMB 4.8 million (Figure 5D). In 2021, the percentage of approved breast imaging AI projects was 11.7% in medical imaging AI and 39.29% in breast imaging (Figure 5E).

In terms of the types of funding for AI projects in breast imaging, they were mainly general programs and young scientist funds, and we still need to work in the direction of “comprehensive coverage and highlighting the key points” (Figure 5F). Among the 29 projects in the field of AI in breast imaging funded by 2021, 15 projects were funded as general programs, accounting for 51.72%; 13 were funded as young scientist funds, accounting for 44.83%; and one was funded by a key program, accounting for 3.45%.

From the perspective of the source provinces of the approved projects (Figure 5G), Guangdong, Shanghai, Tianjin, and Heilongjiang rank among the top, and the main supporting institutions (Figure 5H) include Guangdong Academy of Medical Science, Tianjin Medical University, Fudan University, Harbin Medical University, and Shanghai Jiaotong University. This phenomenon indicates an imbalance in the regional development of breast imaging AI, which is still in its infancy and has not yet been fully popularized.

In summary, with the strong support of the NSFC for medical imaging big data and AI in the past decade, the number of funded projects in this field has been steadily increasing. Regarding the number of applications and funding, AI in breast imaging is still in its early stage. The type of funding is mainly general and youth projects, indicating that the current breast imaging research is still dominated by individual “solo” projects. From the perspective of the source provinces, there are few key programs and talent projects for regional or interdisciplinary cooperation. There is still much room for development in this field. With the further development of technology, the proportion of funding for key programs and talent projects is expected to increase, thereby achieving an overall increase in the quantity‐funding type.

## 6 PROBLEMS AND CHALLENGES OF AI IN BREAST IMAGING

In the past ten years, research on breast AI in China has been booming. As common breast imaging techniques, MG, US, and MRI have outstanding performance in various fields. MG is mostly used for screening. Compared to manual reading, the AI model based on DL can effectively reduce the recall rate and benign biopsy rate of unnecessary BI‐RADS grade 0 lesions without missing high‐grade malignant tumours.^[^ ^40^ ^]^ In addition, AI models can reach the level of senior radiologists in predicting BI‐RADS grade 4 microcalcification.^[^ ^39^ ^]^ US can be widely used in the detection of breast cancer,^[^ ^43^ ^]^ feature characterization,^[^ ^47^ ^–^ ^49^, ^50^ ^]^ and surveillance,^[^ ^54b^ ^]^ especially multimodal US and the combined application of US and MG, which can recognize the molecular subtypes of breast cancer^[^ ^47^, ^48^ ^]^ and assess lymph node involvement effectively.^[^ ^49a,c^ ^]^ MRI is the most sensitive approach for breast cancer detection. Multiomics studies that use AI to combine radiomics with transcriptomics and genomics predict early axillary lymph node metastasis^[^ ^50b^ ^]^ and reveal the heterogeneity of breast cancer^[^ ^51b^ ^]^ from a more microscopic and accurate perspective.

From the perspective of NSFC funding, almost all breast imaging AI projects funded in the past ten years are related to clinical problems, which indicates two aspects. First, from a clinical perspective, although the quality of breast cancer diagnosis and treatment has substantially improved in comparison to the past, there are still many unsolved problems. AI technology may provide an effective method for solving the most challenging clinical problems of breast cancer. Second, from the perspective of AI, the ultimate goal is to solve practical problems in clinical practice. If the technology is detached from actual problems, it is naturally difficult to acquire funding. In addition, from the source provinces and funding types of the approved projects, an imbalance in the regional development of breast imaging AI still exists, which is still in its infancy and has not yet been fully popularized.

Finally, from the perspective of clinical application promotion, although AI is expanding in the field of breast imaging, certain issues need to be solved urgently to promote the coordinated development of breast imaging.

First, a standardized database that can depict the distribution characteristics of breast cancer in the Chinese population should be established. Recently, the Breast Group, Radiology Branch of Chinese Medical Association has published the “Expert Consensus on the Construction and Quality Control of Mammography Database,”^[^ ^57^ ^]^ which aims to guide the construction and quality management of the relevant database of breast imaging AI products and ensure the safe and orderly mining of medical data resources. At present, CMMD is a recognized dataset in China. It includes 1775 patients with a total of 3728 MG images. However, it still has the problem of being a single image source and a small scale. Therefore, it is still necessary to develop the scale of MG datasets and establish a standardized breast US and MRI database.

Second, the transparency of AI algorithms still needs to be promoted. The “black box” issue of AI algorithms is controversial. The opacity not only undermines the confidence of medical decision‐makers but also hinders the sustainable development of AI. Some researchers in the field of computer science have responded to this challenge by developing “explainable AI”, and some scholars in China have conducted a pilot study of a US‐based decision system for breast nodule assessment combined with “explainable AI.”^[^ ^58^ ^]^ However, compared with the number of AI studies that are constantly emerging, research focusing on algorithm transparency is scarce.

Third, it is necessary to combine the research results with the actual medical situation to promote software application and better use it in clinical practice. Currently, the clinical application of breast AI products in China is limited, and they are mainly used for quality control in the image acquisition process and lesion detection. In the future, active cooperation in biomedical engineering will be crucial for translating research into practice.

## 7 CONCLUSION

Breast imaging is at the forefront of medical AI. The establishment of databases and the continuous optimization of DL algorithms have promoted the deepening and advancement of clinical exports of AI technology in image acquisition, screening, feature identification, and surveillance. The NSFC has provided strong support in this direction. However, breast imaging AI, which is still in its early stage, has problems such as a small number of databases, insufficient labelled samples, and inadequate algorithm transparency. However, it is believed that the strong impetus of national funding and discipline distribution, focusing on clinical problems and integrating the efforts of radiologists, model developers, and researchers, will help transform research into practice as soon as possible, promoting AI application to better benefit patients.

## FUNDING INFORMATION

This study was supported by the National Natural Science Foundation of China (No. 82071878 and No. 82271957).

## CONFLICT OF INTEREST STATEMENT

The authors declare no conflicts of interest.
